# Evaluation of the protective role of exogenous growth regulators against Ni toxicity in woody shrub *Daphne jasminea*

**DOI:** 10.1007/s00425-018-2979-6

**Published:** 2018-08-16

**Authors:** Alina Wiszniewska, Ewa Muszyńska, Ewa Hanus-Fajerska, Kinga Dziurka, Michał Dziurka

**Affiliations:** 1 Unit of Botany and Plant Physiology, Institute of Plant Biology and Biotechnology, Faculty of Biotechnology and Horticulture, University of Agriculture in Kraków, Al. 29 Listopada 54, 31-425 Kraków, Poland; 2 Department of Botany, Faculty of Agriculture and Biology, Warsaw University of Life Sciences (SGGW), Nowoursynowska 159, Building 37, 02-776 Warsaw, Poland; 3 The Franciszek Górski Institute of Plant Physiology, Polish Academy of Sciences, Niezapominajek 21, 30-239 Kraków, Poland

**Keywords:** Brassinolide, Gibberellins, Heavy metal, Jasmonic acid, Phytohormones, TEM analysis

## Abstract

**Main conclusion:**

**The results provide a significant verification of the activity of exogenously applied phytohormones: gibberellic acid, jasmonic acid, and brassinolide in the modulation of the plant’s response to nickel treatment.**

**Abstract:**

The study investigated nickel accumulation and its toxicity to *Daphne jasminea* shoots cultured in vitro with or without exogenous supplementation with phytohormones: gibberellic acid (GA3), jasmonic acid (JA), and brassinolide (BL). The aim was to verify the modulatory effect of exogenous plant growth regulators (PGRs) on plant reaction to Ni excess. The combined action of Ni and PGRs was evaluated at the anatomical, ultrastructural, and biochemical levels. Nickel toxicity was manifested in decreased biomass accretion and growth tolerance index (83–53.6%), attributed to enhanced synthesis of growth inhibitors, mainly abscisic acid. As a defence reaction, endogenous gibberellins accumulated. Exogenous GA3 ameliorated the plant reaction to Ni stress, inducing proliferation and growth rate. Ni tolerance in the presence of GA3 was attributed to peroxisomal reactions that stimulated the synthesis of endogenous JA. In contrast, the application of BL caused enhanced Ni accumulation. Plants suffered from pronounced stress due to massive oxidation. The defence strategy of plants subjected to Ni and BL involved cell wall rearrangements. Exogenous JA stimulated the synthesis of active auxins and salicylic acid, contributing to enhanced mitotic activity within explants. However, JA disturbed the integrity of chloroplasts and lamellar compartments. Our study revealed that an action of exogenous PGRs may either enhance tolerance to Ni or increase metal toxicity in *D. jasminea*. Particularly in in vitro culture, where explants are subjected to external phytohormonal stimuli, the combined effects of supplemental PGRs may enhance stress and substantially affect plant development. Our results provide a significant verification of exogenous PGRs activity in the modulation of plant response to nickel.

**Electronic supplementary material:**

The online version of this article (10.1007/s00425-018-2979-6) contains supplementary material, which is available to authorized users.

## Introduction

Nickel (Ni) is a trace metal that, in low concentrations, is essential to plants. Usually, its content in plant tissues ranges from 0.01 to 10 µg/g DW (Seregin and Kozhevnikova 2006; Chen et al. 2009). This element is a constituent of several plant metalloenzymes, including ureases, a few superoxide dismutases, glyoxalases, and hydrogenases (Boer et al. 2014; Fabiano et al. 2015). In recent years, the accumulation of Ni in environment increased due to anthropogenic activities; thus far, more often plants suffer from excess of Ni rather than from its deficiency (Yusuf et al. 2011). Nickel excess is toxic to the majority of plant species, and it causes growth retardation as a result of versatile metabolic disturbances, such as oxidative stress, osmotic and nutrient imbalance, chlorosis and related photosynthesis inhibition, as well as decreased enzymatic activity (Pietrini et al. 2015; Matraszek et al. 2016; Gupta et al. 2017). In natural sites, Ni occurs in high concentrations in ultramafic (serpentine) soils, where local flora developed unique adaptations to cope with elevated level of Ni, resulting in increased tolerance and/or hyperaccumulation. Several species of woody shrubs belonging to the genus *Daphne*, such as *D. mucronata*, *D. gnidium*, and *D. alpina,* exhibit tolerance to high concentrations of nickel (Ater et al. 2000; Bani et al. 2013; Muhammad et al. 2013). Another *Daphne* species, *D. jasminea*, was found to exhibit in vitro tolerance to Pb and Cd (Wiszniewska et al. 2015, 2017a, b). These premises indicate the considerable potential of *Daphne* species to develop stable tolerance against heavy metal toxicity.

Increasing soil pollution with heavy metals forces us to search for effective technologies of soil clean-up, as well as strategies to minimize stress reactions in plants grown in polluted soil (Asgher et al. 2015; Wiszniewska et al. 2016). The latter includes the use of phytohormones and regulatory compounds to increase plant tolerance against heavy metal toxicity (Gangwar et al. 2014). In the case of hyperaccumulating plants, exogenous supplementation with PGRs may facilitate heavy metal accumulation in plant tissues due to enhanced growth and biomass accretion. This, in turn, can accelerate heavy metal removal from the soil via phytoextraction (Cabello-Conejo et al. 2013, 2014; Bulak et al. 2014). In non-hyperaccumulating species, exogenous treatment with PGRs can enhance defence reactions, allowing the restoration of normal growth and development in stressful conditions (Siddiqui et al. 2011; Piotrowska-Niczyporuk et al. 2012).

Plant growth regulators are active components of a signal cascade related to the induction of a plant stress response (Singh et al. 2016). Abiotic stress causes alarming changes in the level of endogenous phytohormones, resulting in growth inhibition to minimize injuries. The usual reaction is a decrease in the concentration of cytokinins, auxins, and gibberellins, and an increase in the content of ABA, jasmonates, and salicylic acid (Atici et al. 2005; Bajguz and Hayat 2009; Bücker-Neto et al. 2017). Other regulatory compounds, such as brassinosteroids, polyamines, peptides, organic acids, and oligosaccharides, also play a role in plant stress response, acting as immunomodulators (Meng et al. 2009; Bajguz 2011; Asgher et al. 2015). It was reported that exogenous application of various PGRs increases plant survival in the presence of heavy metals, mainly by counteracting biomass decrease, enhancing photosynthesis and stimulating antioxidant activity (Saeidi-sar et al. 2007; Piotrowska-Niczyporuk et al. 2012; Gemrotová et al. 2013; Han et al. 2016; Kaur et al. 2018). At the physiological level, these processes are regulated by endogenous phytohormones. In turn, concentrations of these compounds are affected by stress and external hormonal treatment. However, information concerning the effect of exogenously applied PGRs on the profile of endogenous phytohormones in plants suffering from heavy metal stress is still incomplete. There is a little attention given to the fact that supplemental stimuli with PGRs may itself deregulate hormonal balance, contributing to the enhancement of the stress reaction of plants treated with heavy metals.

Therefore, the aim of our study was to assess the level of nickel toxicity to *Daphne jasminea* shoots cultured in vitro in the absence or presence of supplemental PGRs (sPGRs), i.e., gibberellic acid (GA3), jasmonic acid (JA), and brassinolide (BL), and to verify the modulatory effect of exogenous PGRs on plant reactions to Ni excess. We exploited well established, proliferating shoot cultures of a woody plant species to create a model system in which both nickel toxicity and protective role of exogenous PGRs could be verified and evaluated. We hypothesized the following: (1) the presence of Ni induces stress that leads to structural and metabolic rearrangements in plant functioning; (2) the application of different exogenous PGRs affects both the profile of endogenous phytohormones and nickel accumulation, contributing to either the enhancement or suppression of the defence reaction. We evaluated the combined action of Ni and PGRs at the level of organ anatomy and ultrastructure, and biochemical parameters, including targeted profiling of selected endogenous phytohormones, photosynthetic pigments, phenolic compounds, and antioxidant activity. We also compared the effect of sPGRs on Ni accumulation.

## Materials and methods

### Plant cultures

Stock cultures of *Daphne jasminea* (Sibth. & Sm.) shoots were multiplicated on modified WPM medium (Lloyd and McCown 1980) containing 12.3 µM *N*6-[2-isopentyl]adenine (2iP) and 5.37 µM 1-naphthaleneacetic acid (NAA), as described previously (Wiszniewska et al. 2017a, b).

Test cultures were established using 5 mm-long explants. In the first part of an experiment, microcuttings grew on the basal media supplemented with nickel sulfate in concentrations of 0.05, 0.1, 0.5, and 1.0 mM NiSO_4_. The control medium did not contain nickel sulfate and was referred further as Ni(−).

Initially, all sPGRs were pre-tested in concentration of 10 µM. However, in the case of JA and BL, such a high concentration was found to be extremely inhibitory to explants, which virtually did not grow and died within 2 weeks of culture (preliminary study, data not shown). Therefore, in the main experiment, 0.5 µM JA and 0.2 µM BL were applied, corresponding to a concentration of 0.1 mg/L. In contrast, GA3 applied in concentrations of 0.1 mg/L and 1.0 mg/L did not influence the growth of *D. jasminea* microcuttings (preliminary study, not shown), and therefore, 10 µM GA3 (approx. 3.4 mg/L) was tested in the main experiment (according to the literature survey).

In the main experiment, the basal medium containing 0.05 mM NiSO_4_ was enriched with one sPGR: 10 µM GA3 (Sigma); 0.5 µM JA (Sigma), and 0.2 µM BL (Sigma). The control medium without sPGRs contained 0.05 mM NiSO_4_ and was referred further as Ni(+). The pH of the medium was adjusted to 5.6. Nickel sulfate was added to the medium prior to autoclaving at 121 °C, 0.1 MPa for 15 min to avoid alterations in medium pH. All sPGR solutions were filter sterilized using syringe filter (millipore) and added to the warm medium after autoclaving, prior to medium solidification.

Ten microcuttings per 250 mL Erlenmeyer flask were explanted on the respective media. Cultures were maintained for 8 weeks in a growth chamber at 22 °C, under 16 h photoperiod (irradiance 80 μmol m^−2^s^−1^). Cool white fluorescent lamps were used as a light source.

### Growth response and Ni accumulation

After 8 weeks of culture, the obtained shoots were counted and the micropropagation coefficient (MC) was calculated using the formula:

$$ {\text{MC}}  =  ({\text{number of induced adventitious shoots}}/{\text{total number of explants}}).$$

Plant organs were measured and weighted. For dry matter determination, samples were dried at 105 °C in an oven for 24 h and weighted afterwards. Rooting efficiency was expressed as rooting percentage on the basis of number of rooted explants.

Growth tolerance index (GTI, in %) was calculated on the basis of DW of plant organs (either shoots or roots), using the formula:

$$ {\text{GTI}}  =  ({\text{mean DW of organ developed on supplemented medium}}/{\text{mean DW of organ developed on control medium}}) \times 100.$$ 

Plant material for Ni accumulation analysis was washed with distilled water, dried for 24 h in 105 °C, and mineralized in suprapure HNO_3_ (Merck) in a microwave oven. Inductively coupled plasma-mass spectrometry (ICP-MS) (Elan 6100, Perkin Elmer) was applied to determine the Ni content in the entire plantlets.

### Anatomy and ultrastructure

Plant samples were fixed according to Karnovsky (1965), and subsequently post-fixed in 2% OsO_4_ for 2 h at 4 °C, dehydrated in an ethanol series, substituted by propylene oxide and embedded in glycidyl ether 100 epoxy resin (Serva) equivalent to the former Epon812. The resin polymerization was performed at 65 °C for 24 h. Semi-thin sections were prepared using Jung RM 2065 microtome, and stained with methylene blue and azure A prior to examination under a light microscope (Olympus-Provis). Ultra-thin sections were prepared with Ultracut UCT Leica microtome, stained with uranyl acetate and lead citrate, and examined under a transmission electron microscope (Morgagni 268D).

### Biochemical analyses

#### Targeted profiling of endogenous phytohormones

Targeted profiling of endogenous phytohormones was conducted based on method by Dziurka et al. (2016). Plant material was lyophilized and pulverized. To exactly weighted samples of about 10 mg stable isotope labelled internal standards (ISTD), mixture was added. Samples were triple extracted with 1 mL of methanol/water/formic acid buffer (MeOH/H_2_O/HCOOH 15/4/1, by vol.) at ambient temperature. After centrifugation (3 min, 22,000*g*, at 15 °C), clear supernatants were joined and evaporated under N_2_. Residue was suspended in 5% MeOH in 1 M HCOOH, cleaned up on hybrid SPE cartridges (BondElutPlexaPCX, AgilentTechnologies, Santa Clara, CA, USA) and processed as described by Hura et al. (2017). Phytohormones (auxins, cytokinins, gibberellins, abscisic acid, jasmonates, and salicylic acid) were analyzed by ultrahigh-performance liquid chromatography (UHPLC) using an Agilent Infinity 1260 coupled to 6410 Triple Quad LC/MS with ESI (electrospray interface) ion source (Agilent Technologies). Separation was achieved on AscentisExpress RP-Amide analytical column (2.7 μm, 2.1 mm,·150 mm; Supelco, Sigma-Aldrich) at linear gradient of H_2_O vs. acetonitrile with 0.01% of HCOOH, both. Multiple reactions monitoring (MRM) transitions were used for the identification and quantification of all compounds of interest (Supplementary data, Table S1). MassHunter software was used to control the LC–MS/MS system and in data analysis. For MRM parameter optimization, MassHunter Optimizer was used. Internal standard recoveries were used to compensate phytohormone losses occurring at all the stages of analysis. Quantification was based on five-point calibration curves for all standards.

#### Photosynthetic pigments and phenolic compounds

The content of photosynthetic pigments: total chlorophylls (sum of chlorophyll* a* and chlorophyll* b*) and carotenoids in the plant material was determined according to Wellburn (1994) and expressed as mg/g FW of the sample.

Phenolic compounds (total phenols, phenylpropanoids, flavonols, and anthocyanins) were determined using UV/Vis spectrophotometry (Fukumoto and Mazza 2000). Chlorogenic acid (CGA), caffeic acid (CA), and quercetin (QC) were used as standards for total phenolic content (TPC), phenylpropanoids, and flavonols, respectively. Anthocyanin content was expressed as cyanidin (CY), according to its molar extinction. Plant tissue (0.1 g) was ground with 5 cm^3^ of 80% methanol and centrifuged for 15 min at 3000*g*. Supernatant was used for the analysis. The plant extract (0.25 mL) was mixed with 0.25 mL 0.1% HCl (in 96% ethanol) and 4.50 mL 2% HCl (in water), and after 15 min, the absorbances at 280, 320, 360, and 520 nm were read (Hitachi U-2900 spectrophotometer). The content of phenolic compounds was expressed in mg of the respective standard equivalents per 100 g FW.

### Antioxidant activity

Stable free radical DPPH (2,2-diphenyl-1-picrylhydrazyl) was used to test radical scavenging activity of *D. jasminea* plantlets (Pekkarinen et al. 1999). The changes in absorbance of DPPH· solution, following reduction of DPPH, were measured at 517 nm at the moment of extract addition and after 30 min, using a Hitachi U-2900 spectrophotometer. For the analysis, 80% methanol extracts were used. The antioxidant activity of extracts was expressed in % of reduced DPPH· radical by a unit of plant extract.

### Statistical analyses

The experiments were conducted three times (three replicates), with minimum 30 explants (microcuttings) per treatment within one replicate. Data were subjected to ANOVA analysis (Statistica 10.0, StatSoft, Tulsa, OK, USA) and a post hoc Tukey’s test was used to study differences between treatments at *P *< 0.05.

## Results

### Growth inhibition of *D. jasminea* under increasing Ni doses

This part of the study was designed to choose a non-lethal dose of Ni that exerts a toxic effect on cultured plants but allows growth and organogenic response during long-time culturing. As expected, with increasing nickel concentrations, the growth of *D. jasminea* microshoots was gradually inhibited. Application of NiSO_4_ in low doses, i.e., 0.05 mM and 0.1 mM, slightly reduced shoot proliferation, as well as fresh and dry biomass accretion (Table 1). Moreover, in comparison with control treatment without Ni, the growth tolerance index (GTI) decreased to 83–81%, respectively. The micropropagation coefficient (MC) calculated after 8 weeks of culture reached 3.3 for 0.05 mM and 2.7 for 0.1 mM (differences statistically insignificant, *P *> 0.05), in contrast to 4.1 in the absence of Ni (*P *< 0.05) (Table 1). Shoots developed on 0.1 mM and higher concentrations of NiSO_4_ became chlorotic. In the presence of Ni, rooting efficiency also decreased from 70.3% (control) to 57.3 and 53.6% in 0.05 and 0.1 mM NiSO_4_, respectively. Higher doses of Ni, i.e., 0.5 and 1.0 mM NiSO_4_, suppressed rhizogenesis completely. In these treatments, shoots did not proliferate (MC = 1) and elongate, and GTI reached only 48–41% (Table 1).

**Table 1:** Growth parameters of *Daphne jasminea* microplantlets after 8 weeks of culture in the presence of various concentration of nickel sulfate

Nickel sulfate (mM)	MC	Shoot length (mm)	Shoot fresh weight (mg)	Shoot dry biomass (mg)	GTI (%)	Rooted shoots (%)	Root dry biomass (mg)
0	4.1a	16.5 ± 0.7a	134 ± 16a	16 ± 1.2a	n/a	70.3a	7 ± 1.3a
0.05	3.3b	14.1 ± 2.4a	125 ± 6ab	13 ± 1.1b	83.4a	57.3.b	6 ± 0.4a
0.1	2.7b	12.1 ± 2.2b	111 ± 0.024b	13 ± 1.2b	81.7a	53.6b	6 ± 2.1a
0.5	1.0b	11.2 ± 1.6bc	32 ± 13c	6 ± 0.7c	48.4b	0c	–
1.0	1.0b	10.8 ± 1.9c	34 ± 9c	5 ± 1.6c	41.7b	0c	–

*MC* micropropagation coefficient, *GTI* growth tolerance index

Values are means of three replicates ± SD; means indicated by the same letter within the columns do not significantly differ at *P* < 0.05 according to Tukey’s test

### The effect of supplemental PGRs on growth and nickel accumulation

**Fig. 1:**
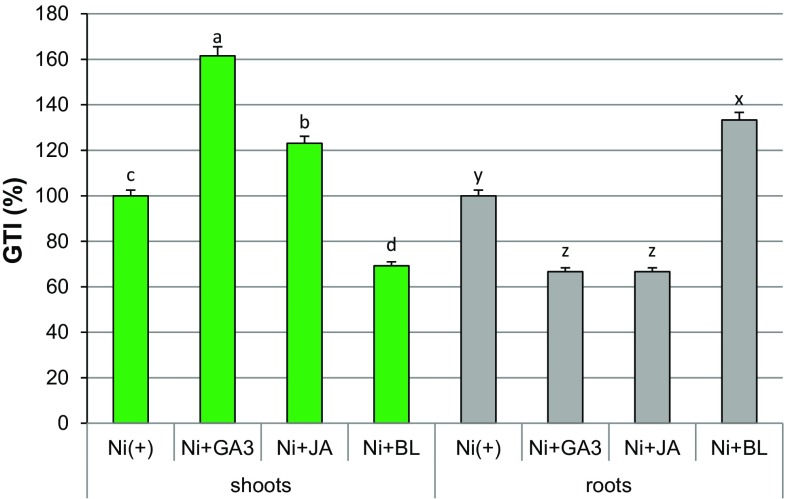
Influence of supplemental PGRs on growth tolerance index (GTI) in *Daphne jasminea* shoots and roots treated with nickel sulfate. Different letters (a–d for shoots; x–z for roots) indicate statistical significance of means (*n *= 3)

On the basis of preliminary screening of the Ni dose (exhibiting relatively the lowest toxicity), in the main experiment, cultures were established on the media containing either 0.05 mM NiSO_4_ alone (control treatment), or in a combination with one of the tested sPGRs. Exogenous sPGRs differentially affected growth responses to Ni ions. A substantial increase in shoot biomass in Ni(+) cultures occurred under supplementation with GA3 and JA, where GTI for shoots amounted to 161.5 and 123.1%, respectively (Table 2; Fig. 1). GA3 particularly stimulated shoot elongation and proliferation, increasing the micropropagation coefficient (MC) to 6.4 (Table 2). The addition of BL slightly enhanced shoot proliferation (MC = 4.9) in comparison with Ni(+) control culture (MC = 3.3); however, shoot elongation was not improved, and biomass accretion decreased (GTI = 69%) (Table 2; Fig. 1).

**Table 2:** Growth parameters of *Daphne jasminea* microplantlets after 8 weeks of culture on media containing nickel sulfate and supplemental PGRs (sPGRs)

Treatment		MC	Shoot length (mm)	Shoot dry biomass (mg)	Rooted shoots (%)	Root dry biomass (mg)
Nickel sulfate (mM)	sPGR (µM)					
0	0	4.1A	16.5 ± 0.7A	16 ± 1.2A	70.3A	7 ± 1.3A
0.05	0	3.3B	14.1 ± 2.4Ab	13 ± 1.1B	57.3B	6 ± 0.4A
0.05	0	3.3d	14.1 ± 2.4b	13 ± 1.1bc	57.3a	6 ± 0.4b
0.05	GA3 10	6.4a	20.7 ± 1.8a	21 ± 2.0a	33.6c	4 ± 0.7c
0.05	JA 0.5	5.7b	17.2 ± 2.2ab	16 ± 0.8b	11.1d	4 ± 0.4c
0.05	BL 0.2	4.9c	8.9 ± 1.3c	9 ± 2.2c	10.2d	8 ± 1.1a

Statistical significance of means for Ni(−) and Ni(+) control cultures is marked with capital letters, while that for Ni(+) cultures without and with sPGRs is marked with lowercase letters

*MC* micropropagation coefficient, *GA* gibberellic acid, *JA* jasmonic acid, *BL* brassinolide

Values are means of three replicates; for each organ, means indicated by the same letter within the columns do not significantly differ at *P* < 0.05 according to Tukey’s test

Rhizogenesis, which had been inhibited by Ni in comparison with untreated control shoots (Table 2), was further suppressed in the presence of sPGRs. Rooting efficiency declined significantly in all the sPGR treatments to 33.6% in Ni + GA3, and only 11.1–10.2% in Ni + JA and Ni + BL, respectively. Interestingly, in Ni + BL-treated cultures, the biomass of adventitious roots increased, and GTI reached the highest value of 133.3% (Table 2; Fig. 1). In Ni + GA3 and Ni + JA cultures, root biomass decreased, and GTI amounted to 61–65% (Fig. 1).

**Fig. 2:**
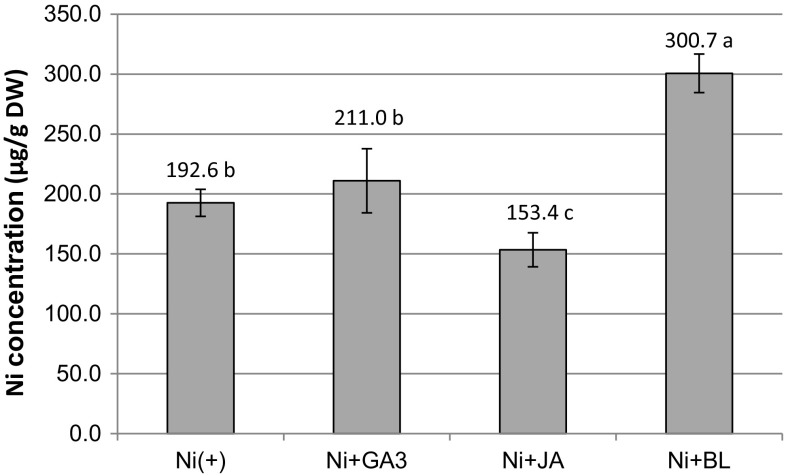
Nickel concentration in *D. jasminea* shoots treated with Ni and supplemental phytohormones

Shoots cultured without nickel (Ni(−)) contained on average 1.7 µg Ni per 1 g DW (data not shown). During culturing in the presence of 0.05 mM NiSO_4_, shoots accumulated considerable amounts of Ni ions, and sPGRs differentially affected this process (Fig. 2). Ni content in Ni(+) shoots amounted to 192.6 µg/g DW. The application of BL increased Ni accumulation to 300.7 µg/g DW, while the application of JA reduced it to 153.4 µg/g DW (*P *< 0.05). GA3 had no influence on Ni accumulation, which had a concentration in Ni + GA3 shoots (211.0 µg/g DW) that was similar to those from Ni(+) control without sPGRs (*P *> 0.05) (Fig. 2).

### Structural response

Evaluation of structural response to Ni and sPGRs treatments was conducted on two distinct organs: (1) shoot bases, which were constantly in contact with culture medium and could be, therefore, directly affected, and (2) leaves, in which potential alterations were results of indirect action of nickel and sPGRs.

**Fig. 3:**
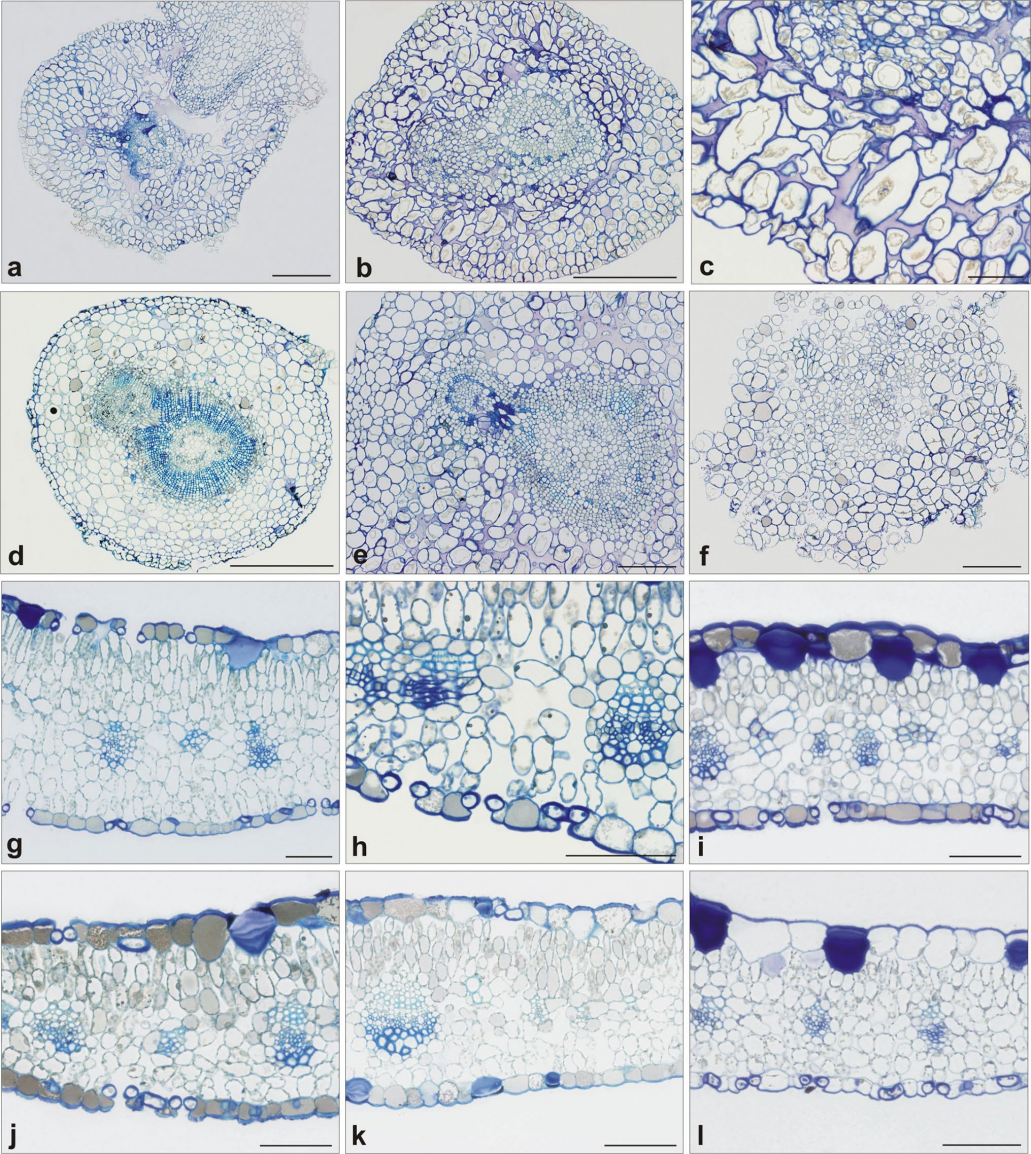
Anatomical structure of *D. jasminea* shoot bases and leaves treated with Ni and supplemental phytohormones. **a**–**f** Shoot bases. Ni(−) (**a**); Ni(+) (**b**, **c**); Ni + GA3 (**d**); Ni + BL (**e**); Ni + JA (**f**). **g**–**l** Leaves. Ni(−) (**g**, **h**); Ni(+) (**i**); Ni + JA (**j**); Ni + BL (**k**); Ni + GA3 (**l**) Bar = 100 µm

Anatomical examination of shoot bases and leaves: Nickel inhibited an organogenic response of cultured explants. The main differences noted between Ni(−) and Ni(+) cultures were delayed differentiation of adventitious shoots, and reduced formation of adventitious roots under Ni exposure. Microplantlets grown without Ni formed adventitious roots internally from the cambial ring (Fig. 3a), while, in all Ni(+) treatments, adventitious roots developed from callus. External cell layers of stems subjected to Ni(+) were disarranged (Fig. 3b, c). Supplemental PGRs affected pattern of organogenic events occurring in the shoot bases. In the presence of GA3 and BL, the formation of adventitious shoots was observed within shoot bases, originating from the cambial ring of the explant stem (Fig. 3d, f). In the presence of JA, stem tissues at the level of shoot base lost their integrity due to dedifferentiation into numerous small division centres (Fig. 3e).

In the leaves of all Ni(+)-treated shoots (including these subjected to sPGRs), palisade parenchyma was less developed than in control Ni(−) leaves (Fig. 3g–l). Especially, in the presence of GA3, the mesophyll was composed mainly of spongy parenchyma. Epidermal cells accumulated a dark substance, presumably phenolics, and this phenomenon was intensified in the presence of JA and BL. In these two cases, phenolic-like compounds were also accumulated in the mesophyll (palisade parenchyma zone) (Fig. 3k, l). In turn, the application of GA3 almost completely suppressed accumulation of phenolics-like compounds in leaf blades (Fig. 3j).

**Fig. 4:**
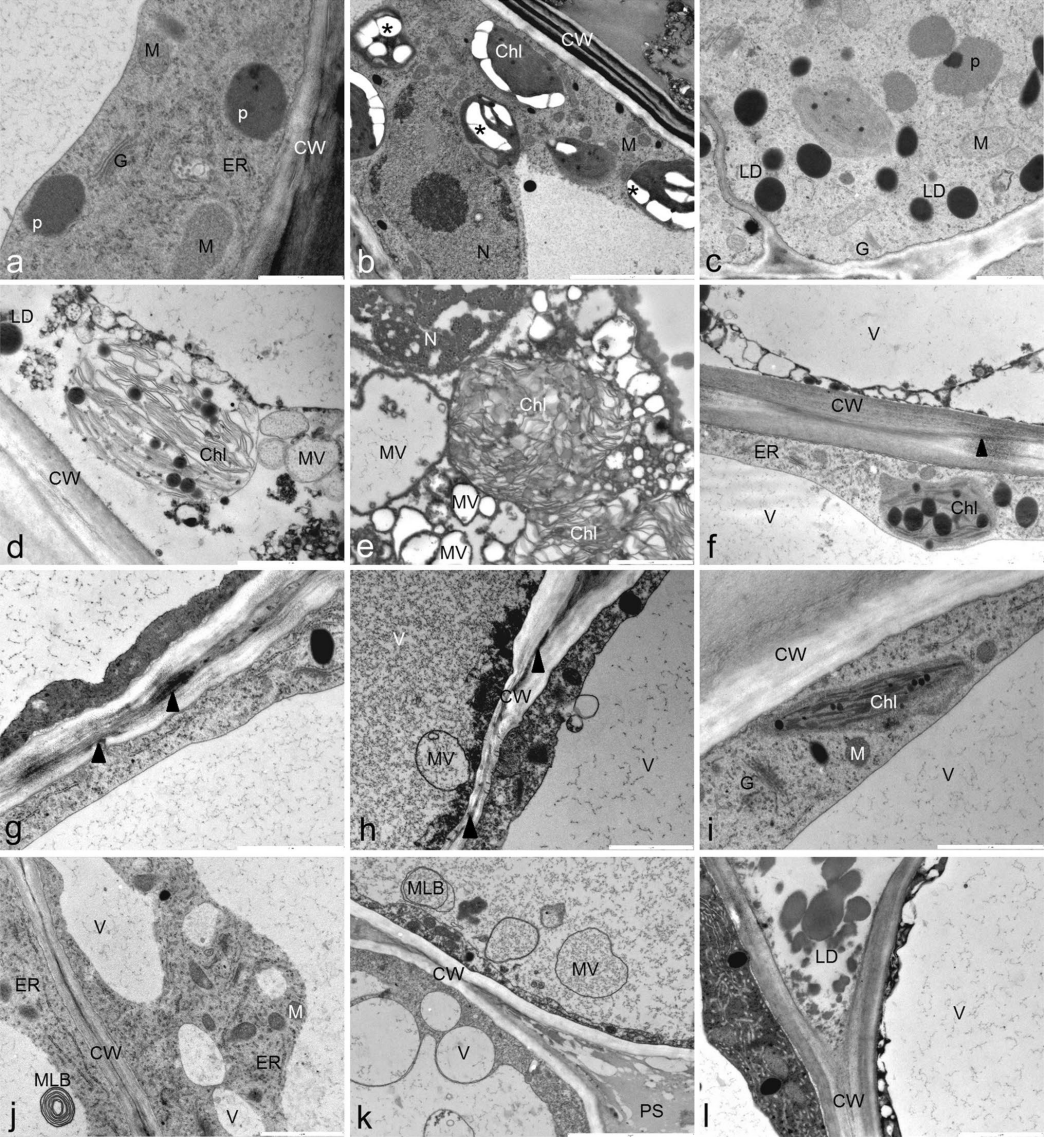
Cell ultrastructure of *D. jasminea* shoot bases treated with Ni and supplemental phytohormones. Ni(−) (**a**–**c**); Ni(+) (**d**–**f**); Ni + JA (**g**, **i**, **l**); Ni + BL (**h**, **k**); Ni + GA3 (**j**). *CW* cell wall, *N* nucleus, *G* golgi apparatus, *V* vacuole, *M* mitochondrion, *asterisk* (*) starch, *Chl* chloroplast, *p* peroxisome, *PS* pectin-like substance, *ER* endoplasmatic reticulum, *triangle* (▲) granular deposits, *LD* lipid droplet, *MLB* multilamellar body, *MV* microvesicle

Ultrastructure of shoot bases and leaves: cells of the shoot base in control Ni(−) treatment had a thick layer of cytoplasm rich in mitochondria, endoplasmatic reticulum, and other organelles (Fig. 4a–c). Under Ni exposure, disturbances in cell ultrastructure occurred. Most of Ni-treated cells had disintegrated cytoplasm with microvesicles and deformed chloroplasts (Fig. 4d–f). In the cells with well-preserved cytoplasm, numerous ER and Golgi apparatus appeared (Fig. 4f). Within cell walls, granular deposits were accumulated (Fig. 4f). In contrast to Ni(+), in Ni + sPGRs, damaged cells were present mostly in the external part of the stem. In cells treated with Ni + JA and Ni + BL, microscopic channels with granular deposits were formed in the cell walls (Fig. 4g, h). In the inner layers of sPGR-treated shoots, cell degeneration was less advanced (Fig. 4i). Disturbances included intensified vacuolation and the formation of multilamellar bodies inside the vacuoles (Fig. 4j, k). Furthermore, independently of the position within the shoot base, cells treated with Ni + JA and Ni + BL were surrounded by pectin-like substances and lipid droplets (Fig. 4k, l).

**Fig. 5:**
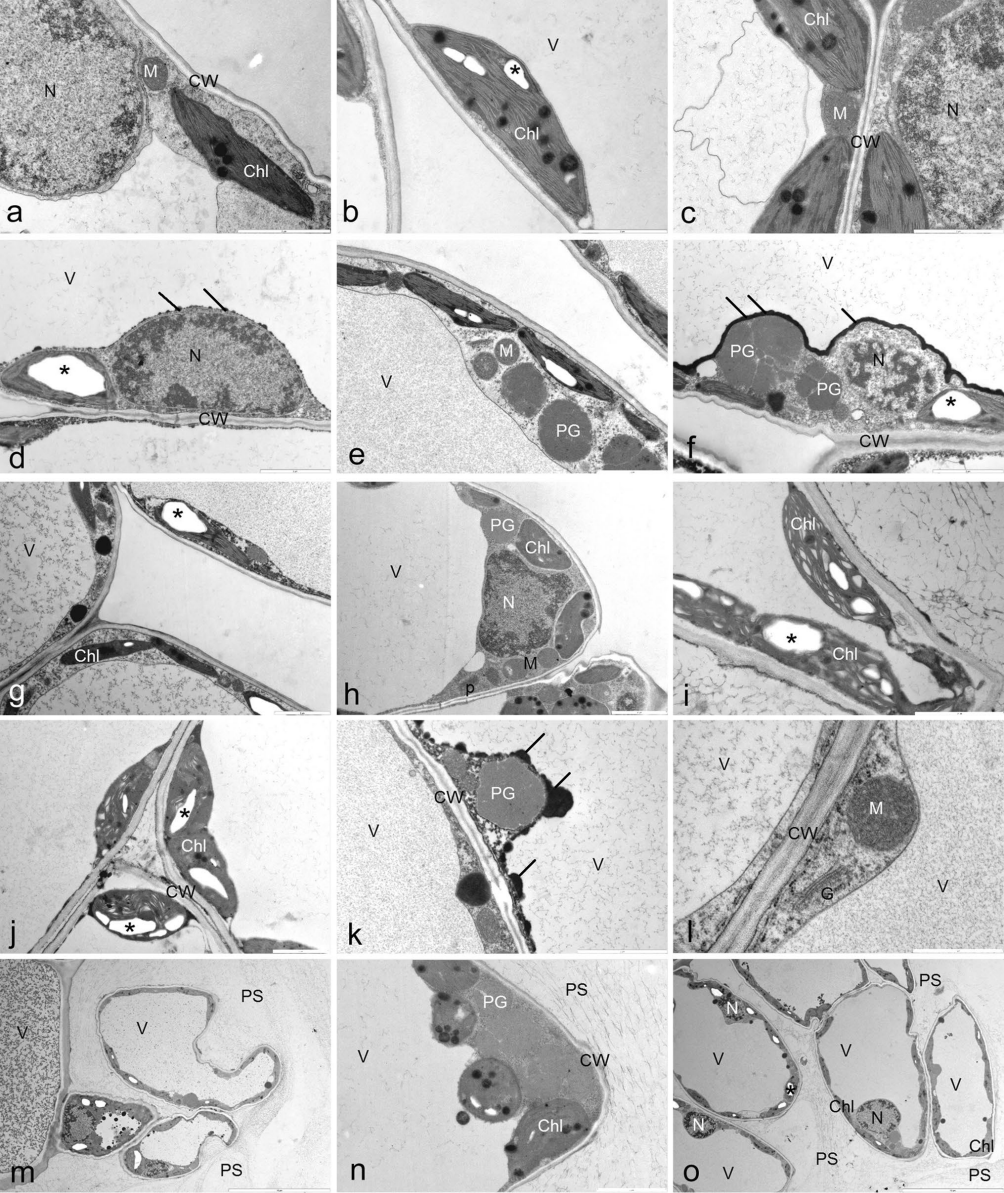
Cell ultrastructure of *D. jasminea* leaves treated with Ni and supplemental phytohormones. Ni(−) (**a**–**c**); Ni(+) (**d**, **m**); Ni + BL (**e**, **i**, **j**, **l**); Ni + JA (**k**); Ni + GA3 (**h**, **n**). *CW* cell wall, *N* nucleus, *G* golgi apparatus, *V* vacuole, *M* mitochondrion, *asterisk* (*) starch, *Chl* chloroplast, *line* (▬) phenolic-like compounds, *PG* protein grain, *p* peroxisome, *PS* pectin-like substance

Cells of leaves from control Ni(−) medium were typically arranged, with normally formed plastids, mitochondria, endoplasmic reticulum, Golgi apparatus, peroxisomes, and vacuoles with smooth tonoplast (Fig. 5a–c). Chloroplasts had a regular structure with numerous grana and plastoglobuli in spongy parenchyma or with a few small starch grains in palisade cells (Fig. 5b). In turn, leaf cells of all Ni(+)-treated shoots accumulated starch grains and protein deposits in the external layer of the palisade parenchyma (Fig. 5d–f). In addition, in palisade cells of leaves treated with Ni(+) alone, Ni + JA electron-dense deposits of phenolic-like compounds were formed either on the tonoplast or within the cytoplasm (Fig. 5d, f, g, k). In cells from sPGRs treatments, irrespectively of the PGR type, numerous round mitochondria with areas of lower electron density were present (Fig. 5h, l). Ni + GA3 cells were particularly rich in peroxisomes (Fig. 5h). The ultrastructure of chloroplasts was altered under Ni exposure. Especially, in palisade cells from Ni(+), Ni + GA3 and Ni + BL, thylakoids and grana had a looser arrangement, while, in spongy cells, swollen stroma and dilated thylakoids were observed (Fig. 5h–j). Moreover, in Ni(+), Ni + GA3 and Ni + BL treatments, some parts of cell walls excreted a pectin-like substance into the intercellular space (Fig. 5i, m–o).

### Biochemical response

#### Profile of endogenous phytohormones

We have separately analyzed differences between the profile of endogenous PGRs in control cultures untreated and treated with Ni and between Ni(+)-treatments with without and with sPGRs. For the main classes of endogenous PGRs, we determined the level of both active and deactivated compounds and, in the case of cytokinins and gibberellins, also intermediate compounds (Table 3).

**Table 3:** Profile of endogenous phytohormones in *D. jasminea* organs treated with nickel sulfate and supplemental phytohormones

Treatment			Endogenous phytohormones (nmol/g d.w.)												
Code	NiSO_4_ (mM)	Supplemental phytohormones	Auxins			Cytokinins			Gibberellins			Abscisic acid		Jasmonates	Salicylic acid
			Actv	Deact		Actv	Inter	Deact	Actv	Inter	Deact	Actv	Deact		
				Oxo	Conj										
Shoots															
Ni(−)	0	0	0.58A	0.00A	4.78A	0.41A	0.04A	0.04A	0.83A	1.27A	0.72B	0.83A	2.33B	0.31B	15.1A
Ni(+)	0.05	0	0.64A	0.01B	3.69B	0.37A	0.05A	0.04A	1.08A	1.19A	1.34A	1.09A	3.63A	0.40A	7.1B
Ni(+)	0.05	0	0.64c	0.01b	3.69b	0.37a	0.05a	0.04a	1.08b	1.19a	1.34a	1.09c	3.63a	0.40b	7.1d
Ni + GA3	0.05	GA3	0.79b	0.01b	6.03a	0.37a	0.05a	0.05a	2.06a	1.03a	1.53a	1.60c	3.52a	0.52a	19.2a
Ni + JA	0.05	JA	1.36a	0.05a	2.00c	0.27b	0.06a	0.03a	0.49c	1.52a	1.03a	4.65b	0.16b	0.29b	13.5b
Ni + BL	0.05	BL	0.81b	0.04a	2.85bc	0.21b	0.06a	0.04a	0.57c	0.92a	0.87ab	11.74a	0.22b	0.25b	9.9c
Roots															
Ni(−)	0	0	0.79B	0.31A	8.64B	0.82B	0.06B	0.06B	1.68B	1.57A	0.51B	8.43A	14.67B	4.85A	11.5B
Ni(+)	0.05	0	2.67A	0.28A	14.41A	2.60A	0.15A	0.42A	3.13A	1.62A	2.21A	8.35A	26.45A	0.64B	14.9A
Ni(+)	0.05	0	2.67a	0.28b	14.41a	2.60a	0.15b	0.42a	3.13c	1.62b	2.21b	8.35b	26.45a	0.64b	14.9a
Ni + GA3	0.05	GA3	1.35b	0.00d	9.19b	1.61b	0.12b	0.27b	7.82a	0.99c	0.00d	8.30b	25.42a	1.03a	9.6b
Ni + JA	0.05	JA	0.98c	0.09c	2.26c	0.56c	0.11b	0.07c	1.03d	2.26b	0.84c	2.08c	0.57b	0.21c	4.3c
Ni + BL	0.05	BL	2.62a	0.52a	7.70b	2.88a	0.40a	0.43a	4.78b	9.03a	3.73a	10.63a	0.30b	0.54b	14.6a

10 µM gibberellic acid; 0.5 µM jasmonic acid; 0.2 µM brassinolide; active compounds; deactivated compounds; oxidized compounds; conjugates; intermediate compounds

Values are means of three replicates; for each organ, means indicated by the same letter within the columns do not significantly differ at *P* < 0.05 according to Tukey’s test. Statistical significance of means for Ni(−) and Ni(+) control cultures is marked with capital letters, while that for Ni(+) cultures without and with sPGRs is marked with lowercase letters

Endogenous phytohormones in Ni(−) and Ni(+) cultures: the profiles of endogenous PGRs differed substantially between shoots and roots. In the shoots, the contents of active forms of auxins, cytokinins, gibberellins, abscisic acid, and jasmonates were virtually the same in Ni(−) and Ni(+) treatments. As a reaction to Ni, an increase occurred in the level of inactive forms of abscisic acid, oxidized auxins, as well as inactive gibberellins. In turn, the level of salicylic acid and conjugated (inactive) auxins decreased (Table 3).

In the Ni(+) roots, a significant increase occurred in the content of active forms of auxins, cytokinins, gibberellins, and salicylic acid in comparison with Ni(−) roots (Table 3). Among deactivated auxins, the concentration of conjugated compounds increased under Ni exposure, and the level of oxidized compounds did not differ between Ni(−) and Ni(+) roots. The level of both intermediate and deactivated cytokinins increased, and endogenous jasmonates decreased (Table 3). Under Ni exposure, the content of active abscisic acid was not altered; however, inactive ABA glucosyl ester accumulated.

Endogenous phytohormones in Ni(+) cultures without and with sPGRs: In the case of shoots, medium supplementation with exogenous GA3 led to the increase in endogenous auxins, especially their conjugates, active gibberellins, jasmonates, and salicylic acid (Table 3). Application of JA increased the content of active auxins, salicylic acid, and an active form of ABA. The highest increase in active ABA content occurred in the Ni + BL medium. Both JA and BL reduced the concentration of active cytokinins and gibberellins, with a constant proportion of intermediate and deactivated compounds. In addition, these two sPGRs increased the level of oxidized auxins and simultaneously decreased the content of conjugated forms (Table 3).

In comparison with roots developed on Ni(+) medium without sPGRs, in the presence of sPGRs, the content of auxin conjugates was reduced (Table 3). Apart from that, each sPGR differentially modulated the level of endogenous PGRs (Table 3). Medium supplementation with GA3 increased the content of active gibberellins and jasmonates. In the presence of GA3 and JA, the concentrations of active and oxidized auxins decreased. JA reduced the content of cytokinins in the roots, especially active and deactivated compounds, as well as free ABA, endogenous jasmonates, and salicylic acid. BL enhanced auxin oxidation, synthesis of cytokinin intermediates, all forms of gibberellins, and an active form of ABA (Table 3). In turn, BL did not affect the content of jasmonates and salicylic acid.

#### Pigments, phenolic compounds, and radical scavenging activity

**Fig. 6:**
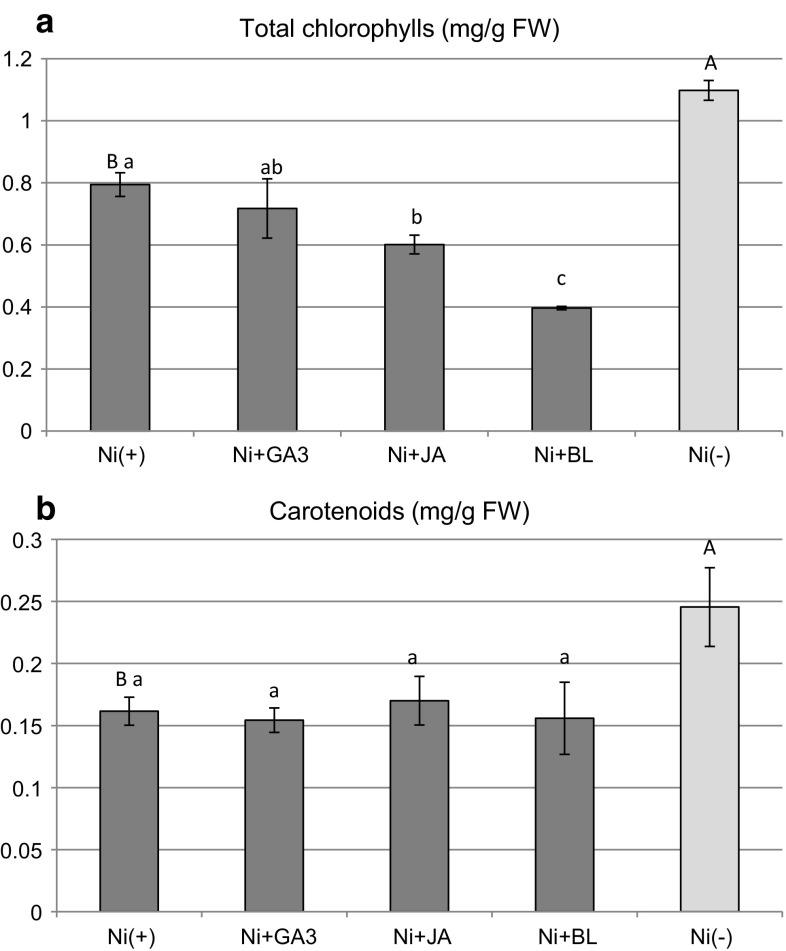
Content of photosynthetic pigments in *D. jasminea* shoots treated with Ni and supplemental phytohormones. Capital letters indicate statistical significance of means (*n *= 4) for Ni(−) and Ni(+) treatments. Lowercase letters indicate statistical significance of means (*n *= 4) for Ni(+) and Ni + sPGRs treatments

Toxicity of Ni to cultured explants was manifested in the decreased synthesis of photosynthetic pigments, both chlorophylls and carotenoids, in comparison with Ni(−) treatment (Fig. 6). In Ni(+) cultures, the application of exogenous phytohormones did not ameliorate pigment accumulation. Total chlorophyll content decreased significantly in Ni + JA and Ni + BL, while, in the case of Ni + GA3, it was the same as in Ni(+) cultures without sPGRs (Fig. 6). No influence of applied sPGRs was observed on the content of carotenoids (Fig. 6).

**Fig. 7:**
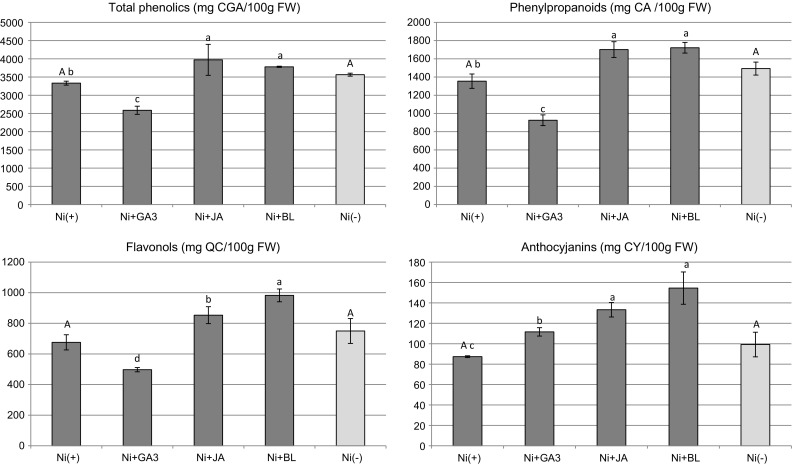
Profile of phenolic compounds in *D. jasminea* shoots treated with Ni and supplemental phytohormones. Capital letters indicate statistical significance of means (*n *= 4) for Ni(−) and Ni(+) treatments. Lowercase letters indicate statistical significance of means (*n *= 4) for Ni(+) and Ni + sPGRs treatments *CGA* chlorogenic acid,* CA* caffeic acid,* QC* quercetin,* CY* cyanidin

Considering the phenolic profile of Ni-treated shoots, the content of total phenolics, phenylpropanoids, and flavonols significantly increased in Ni + JA and Ni + BL, in comparison with Ni(+) treatment without sPGRs (Fig. 7). In contrast, the accumulation of these compounds was significantly reduced in shoots from Ni + GA3 medium (Fig. 7). In Ni(+) cultures, all applied sPGRs caused a substantial increase in the anthocyanin content. When compared with Ni(−) cultures, the presence of Ni did not enhance the accumulation of phenolic compounds.

**Fig. 8:**
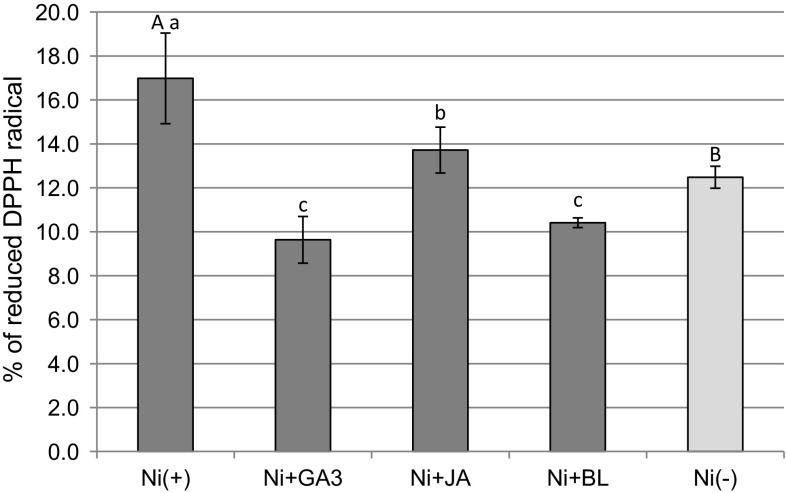
Radical scavenging activity in *D. jasminea* shoots treated with Ni and supplemental phytohormones. Capital letters indicate statistical significance of means (*n *= 4) for Ni(−) and Ni(+) treatments. Lowercase letters indicate statistical significance of means (*n *= 4) for Ni(+) and Ni + sPGRs treatments. DPPH, 2,2-diphenyl-1-picrylhydrazyl

Antioxidant capacity of shoot tissues, measured as an efficiency of radical scavenging, was elevated in the presence of Ni (Fig. 8). Medium supplementation with sPGRs, especially GA3 and BL, caused a significant reduction in radical scavenging activity (RSA). Comparing sPGR treatments, the highest radical scavenging activity occurred in Ni + JA (Fig. 8).

## Discussion

### The effects of Ni stress in *D. jasminea* microplantlets

Toxicity of Ni to plants was attributed to detrimental effects caused by water, osmotic, and oxidative stresses (Yusuf et al. 2011; Gupta et al. 2017). In our study on *D. jasminea* toxicity, increasing Ni concentrations were manifested by inhibited growth, proliferation, and organogenesis. After the initial screening for an optimal, non-lethal Ni level to be applied in shoot cultures, we have evaluated structural and physiological responses to toxic ions.

Our study revealed organ-specific alterations in the content of endogenous phytohormones in *D. jasminea* exposed to Ni. Analysis of these alterations allows, at least partially, defining mechanisms of nickel toxicity and plant defence reaction. Interestingly, in neither shoots nor roots, enhanced accumulation of free ABA occurred, which is the opposite to the content of ABA glucosyl ester (ABA-GE), an inactive form of ABA. Enhanced synthesis of free ABA is the most common reaction in plants subjected to abiotic stress, such as drought and cold (Peleg and Blumwald 2011). Moreover, heavy metals, including Ni, were reported to cause disturbances in the water status that results in enhanced ABA synthesis (Kim et al. 2014; Vishwakarma et al. 2017). This compound regulates stomata closure in stressed plants, regulating transpiration in unfavourable conditions. However, in our experiment, which was conducted in in vitro culture, toxic effects were unlikely related to water deficiency. The content of free ABA was not altered under Ni exposure, indicating sufficient water availability. This was confirmed by the anatomical survey, which revealed that *D. jasminea* leaves had opened stomata distributed in both the lower and upper epidermis. On the other hand, the accumulation of ABA-GE, especially pronounced in the roots, suggests the involvement of ABA in Ni stress response. ABA glucosyl ester is regarded as a storage and transport form of ABA and a root-to-shoot signalling molecule specific to osmotic stress (Piotrowska-Niczyporuk and Bajguz 2011; Burla et al. 2013; Bücker-Neto et al. 2017). Therefore, nickel toxicity in *D. jasminea* can be related to osmotic imbalance that was manifested in enhanced accumulation of ABA-GE. Osmotic instability was associated with a reduction in biomass accretion, as in in vitro cultured *Hypericum perforatum* (Cui et al. 2010).

In *D. jasminea* shoots, the stress reaction was also manifested by the oxidation of auxins, the accumulation of jasmonates, and the reduction of the salicylic acid content. Oxidation, unlike conjugation, is a way of irreversible inactivation of auxins. It is also linked with elevated ROS production during oxidative stress (Jouili et al. 2011; Peer et al. 2013). We have, therefore, analyzed some aspects of the antioxidant response of Ni-treated shoots. Although radical scavenging activity increased, the content of phenolic compounds was virtually unaffected. However, there is an interesting relationship between the content of oxidized auxins and flavonols. The latter are regarded as buffers of cellular ROS levels, and their content is usually negatively correlated with the content of oxidized auxins (Peer et al. 2013). In *D. jasminea,* enhanced production of cellular ROS under Ni exposure could be attributed to the signal role of ROS generated in the presence of a low dose of nickel rather than to the induction of massive oxidative stress.

Enhanced accumulation of endogenous jasmonates is associated with the toxic action of heavy metals (Gallego et al. 2012). Heavy metals cause the degradation of cellular membranes and the induction of an octodecanoic pathway, leading to synthesis of jasmonates (Maksymiec et al. 2005). As revealed by TEM, in cells of *D. jasminea,* membrane degradation occurred, especially the disintegration of chloroplasts and formation of microvesicles. Moreover, the content of pigments bound to membranes, i.e., chlorophylls and carotenoids, substantially decreased. We can, therefore, assume that the increased accumulation of jasmonates was a consequence of the nickel-induced degradation of cellular membranes.

Interestingly, under Ni stress, *Daphne* plants were able to maintain (in shoots) or even elevate (in roots) the content of active forms of growth promoters, namely auxins, gibberellins, and cytokinins. Usually, together with increase in ABA content, the level of other endogenous PGRs, especially cytokinins, decreases (Peleg and Blumwald 2011; Piotrowska-Niczyporuk et al. 2012). Reaction in *Daphne* cultures can be attributed to the hormetic dose effect, in which low doses of toxic agent stimulate the synthesis of growth promoters as a defence reaction (Calabrese and Blain 2009).

### Modulatory role of exogenous growth regulators in Ni toxicity

At the structural level, the most pronounced effect of sPGR application was a limited cellular damage in the inner parts of shoot bases. Shoots developed in Ni(+) without sPGRs suffered from serious cellular disorganization, mainly the formation of microvesicles and multilamellar bodies, suggesting autophagy (van Doorn et al. 2015), and chloroplast degradation. Such alterations were also apparent in sPGR-treated shoots, but they were limited to the cells of external layers. Inner cells maintained an undisturbed structure, divided, and formed adventitious shoots.

All phytohormones applied in this study are widely reported to increase plant tolerance to heavy metal stress (He et al. 2015; Sirhindi et al. 2016; Singh and Prasad 2017). In *D. jasminea,* exogenous application of sPGRs altered a reaction of propagated shoots to nickel treatment. The most surprising was the fact that BL inhibited shoot proliferation and growth. Recent reports highlight the metal stress-ameliorating role of various brassinosteroids, including BL (Sharma et al. 2008; Singh and Prasad 2017). Growth inhibition of *D. jasminea* could be the result of enhanced accumulations of Ni in the presence of BL. In tolerant plants, Ni is chelated and accumulated in vacuoles, while, in non-tolerant plants, it is rather stored in cytoplasm and cell walls (Krämer et al. 2000). In *D. jasminea* cells of shoots accumulating higher amounts of Ni, namely from Ni(+) and Ni^+^ BL media, we observed substantial alterations in the cell wall structure. The most prominent was the presence of granular metal deposits in the cell wall. In addition, in Ni + BL, the formation of cell wall channels with granular deposits and secretion of lipids and pectins occurred. Accumulation of waxes and a pectin layer can be considered a defence reaction aimed at restricting the transport of heavy metals, such as Pb or Ni, into the protoplast (Krzesłowska et al. 2009, 2016; Meychik et al. 2014). Therefore, *D. jasminea* can be classified as Ni-sensitive species, counteracting Ni toxicity by cell wall reorganization. We noted a strong stress reaction in microplantlets cultured in Ni + BL medium, manifested by the increased synthesis of ABA and anthocyanins, as well as an elevated auxin oxidation with a simultaneous decline of radical scavenging activity. The observed stress response indicates that, in the applied culture system, the action of BL enhanced Ni accumulation to a toxic level. The phenomenon of increased Ni accumulation in the presence of BL is discussed below in the following subsection.

GA3 and JA applied to Ni(+) medium restored the capacity of shoot proliferation and biomass accretion to the level observed in untreated shoots. Gibberellic acid substantially ameliorated the growth and physiological condition of *D. jasminea*, which was reflected in the phytohormonal profile. The high level of exogenous GA3 enhanced shoot organogenesis by the stimulation of the synthesis of active forms of gibberellins and auxins, as well as auxin conjugates. The absence of oxidized auxins in the roots and their low level in the shoots indicated the repression of auxin deactivation in the presence of GA3. Interestingly, as revealed by extract analysis and anatomical survey, the accumulation of phenolic compounds was significantly inhibited. There is evidence that exogenous gibberellins may reduce the content of phenolic compounds in plant tissues and interplay with antioxidant capacity (Tian et al. 2011). Therefore, we can assume that non-enzymatic antioxidants were not likely involved in antioxidant response of *D. jasminea* subjected to Ni + GA3. On the other hand, in leaf cells from this treatment, numerous peroxisomes were present, suggesting that some oxidative processes were stimulated. It can be related to the substantial accumulation of endogenous JA in the presence of GA3, since JA production is carried out in peroxisomes via controlled β-oxidation (León 2013). The increased content of endogenous jasmonates is often associated with enhanced tolerance to heavy metal stress (Maksymiec et al. 2005; Yan et al. 2013). This supports our finding that supplemental application of GA3 contributed to higher Ni tolerance in *D. jasminea*. In this case, the mechanism responsible for enhanced Ni tolerance may be related to balanced phytohormone synthesis, namely the production of endogenous JA and metabolically active growth promoters (auxins and gibberellins).

Exogenous JA also ameliorated multiplication and growth parameters of *D. jasminea* shoots under Ni exposure. This reaction could be related to enhanced synthesis of active auxins and salicylic acid. As reported by Della Rovere et al. (2013), the meristematic capacity of cells is induced by auxin accumulation. The observed formation of numerous meristematic centres in Ni + JA-treated stem bases could be a result of the stimulation of auxin accumulation in the shoots. The amelioration of growth response could also be attributed to enhanced antioxidant activity. Jasmonates are known to mitigate oxidative stress through the induction of antioxidant components, both enzymatic and non-enzymatic (Farooq et al. 2016; Sirhindi et al. 2016). In our study, the increased antioxidant response after Ni + JA treatment was manifested by a higher content of phenolic compounds and a considerably high radical scavenging activity (in comparison with other sPGRs). On the other hand, an increased content of endogenous ABA and the decreased accumulation of gibberellins suggest that exogenous JA was also involved in the processes of inhibitory character. Stress reactions were unlikely related only to Ni accumulation, since this process was significantly restricted. The main reason of stress reaction in Ni + JA-treated plants seemed to be the enhanced digestion of cellular components. At the ultrastructural level, the formation of vesicles and multilamellar bodies as well as the disorganization of the chloroplast structure were observed. It was recently found that exogenous jasmonates may activate the chlorophyll degradation machinery (Zhu et al. 2015). Rapid decline in the content of chlorophyll supports this explanation of stress reaction in Ni + JA-treated *D. jasminea*.

### Effect of sPGRs on Ni accumulation in cultured shoots

The use of sPGRs was also applied for enhancing Ni uptake by cultured shoots. It was surprising to reveal that only BL enhanced Ni accumulation in *D. jasminea*. The uptake of heavy metals usually decreases when plants are treated with stress hormones due to restricted transpiration and stomatal closure (Bücker-Neto et al. 2017). In general, brassinosteroids influence the redistribution of metals between shoots and roots (Wang et al. 2015; Waisi et al. 2017). However, despite the fact that these compounds reduce metal translocation from roots to shoots, preferential binding of some heavy metals in the roots may occur (Sharma et al. 2008; Singh and Prasad 2017; Xu et al. 2018). On the other hand, there are single reports on the promoting effect of BL on Ni translocation, such as in the hyperaccumulator *Solanum nigrum* (Soares et al. 2016). This phenomenon was attributed to the mechanism aimed at the protection of roots. In our study, an explanation of enhanced Ni accumulation may be simpler, because, although *D. jasminea* cultured in vitro rooted poorly in the presence of Ni and BL, Ni could be absorbed directly by the shoot base immersed in the culture medium. The way that BL promoted direct Ni uptake remains still to be construed. As these compounds are not likely involved in long-distance transport (Lacombe and Achard 2016), the observed reaction could be related to phenomena existing in the intact zone between the shoot base and the culture medium; for instance, cell wall rearrangements or synthesis of organic acids of metal-chelating properties.

It was reported that application of brassinosteroids exerts different effects on metal accumulation in plants, either increasing or decreasing their uptake depending on the applied form of BL, its concentration, or type of metal ion (Sharma et al. 2008; Xu et al. 2018). Therefore, further research is needed to clarify the role of BL in Ni accumulation and in *D. jasminea* response to metal stress.

The exogenous application of GA3 did not affect Ni accumulation in *D. jasminea* plantlets, compared to non-sPGRs Ni(+) treatment. Similarly, Meng et al. (2009) did not observe any influence of exogenous GA3 on internal Cd accumulation in *Brassica napus*. This reaction was rather unusual, since GA3 was reported to be involved in alteration of the membrane permeability and regulation of membrane transport processes, leading to a decrease in metal uptake (Rubio et al. 1994; Moya et al. 1995). This reduction of Ni accumulation in GA3-treated plants was observed in Ni-hyperaccumulating species of *Alyssum* and *Noccaea* (Cabello-Conejo et al. 2014) or non-accumulating specimens of *Vigna radiata* (Ali et al. 2015).

It was found that JA and its derivative, methyl jasmonate (MeJA), significantly inhibit the uptake of heavy metals by the reduction in transpiration rate (Piotrowska et al. 2009; Chen et al. 2014). In our study, the decreased Ni concentration in *D. jasminea* shoots was unlikely related to a disturbed water balance, since the experiment was held in high humidity of in vitro culture and leaf stomata were opened. The uptake of Ni could be repressed by an increased concentration of phenolic compounds in *D. jasminea* shoots treated with Ni + JA. The relationship between heavy metal accumulation and the synthesis of phenolic compounds could be in agreement with the findings of Kováčik et al. (2011) and Chen et al. (2014). They concluded that exogenous application of MeJA inhibits Ni, Cd, and As uptake by enhanced accumulation of phenolics in *Matricaria chamomilla* and *Kandelia obovata* plants.

## Conclusions

Nickel toxicity to *Daphne jasminea* was attributed to enhanced synthesis of growth inhibitors, mainly ABA, in cultured shoots. Medium supplementation with exogenous GA3, JA, and BL was applied as a strategy to ameliorate plant growth and to stimulate the defence system under unfavourable conditions. The tested sPGRs differentially modulated the plant response, activating various defence pathways, from the stimulation of peroxisomal reactions for increased JA accumulation, via synthesis of endogenous growth promoters, such as active auxins and salicylic acid, to cell wall rearrangements. Our main message is that the action of exogenous PGRs may either increase plant tolerance to Ni stress, or elevate the stress itself, especially when an accumulation of toxic metal is facilitated. In in vitro culture with explants subjected to external phytohormonal stimuli, the combined effects of supplemental compounds may modulate plant growth and tolerance levels.

### Author contribution statement

Conception and design: AW and EHF. Analysis and interpretation of the data: AW, EM, KD, and MD. Drafting of the article: AW and EM. Critical revision of the article for important intellectual content: AW and EHF. Final approval of the article: AW, EM, EHF, KD, and MD. Statistical expertise: AW, KD, and MD. Obtaining of funding: AW and EHF. Collection and assembly of data: AW, EM, KD, and MD.

## Electronic supplementary material

Below is the link to the electronic supplementary material.

Supplementary material 1 (DOCX 23 kb)

## Abbreviations

BL: Brassinolide
PGRs: Plant growth regulators
sPGRs: Supplemental plant growth regulators
JA: Jasmonic acid
GA3: Gibberellic acid
ROS: Reactive oxygen species
TEM: Transmission electron microscopy
