# Supplementary material for: Evaluation of the protective role of exogenous growth regulators against Ni toxicity in woody shrub Daphne jasminea
Source: Planta. 2018 Aug 16;248(6):1365–81. doi: 10.1007/s00425-018-2979-6 (PMC6244662; doi:10.1007/s00425-018-2979-6)
Supplement: Supplementary file 1 — Supplementary material 1 (DOCX 23 kb) [file 425_2018_2979_MOESM1_ESM.docx]

**Table S1** Multiple reactions monitoring (MRM) transitions for the analyzed phytohormones at: positive ion mode (+ESI), capillary voltage 4 kV, gas temperature 350 °C, gas flow 12 l/min and nebulizer pressure 35 psi.

| Compound | Type of ion | Quantifier transition  (precursor/product ions) | Fragmentor voltage (V) | Collision energy (V) | MRM Start Time (min) |
| --- | --- | --- | --- | --- | --- |
| t-Z7G | [M+H]^+^ | 382.1/220.1 | 122 | 17 | 1.6 |
| t-ZOG | [M+H]^+^ | 382.1/202.1 | 142 | 17 |  |
| t-Z | [M+H]^+^ | 220.2/136.3 | 85 | 9 |  |
| c-Z | [M+H]^+^ | 220.2/136.3 | 85 | 9 |  |
| DHZ-N15 | [M+H]^+^ | 226.2/140 | 124 | 18 |  |
| DHZ | [M+H]^+^ | 222.2/136 | 124 | 18 |  |
| K-N15 | [M+H]^+^ | 220.1/192.3 | 90 | 9 | 4.0 |
| K | [M+H]^+^ | 216.1/188.3 | 90 | 9 |  |
| oxIAA | [M+H]^+^ | 192.2/146.1 | 54 | 9 |  |
| GA8 | [M-H_2_O+H]^+^ | 319.3/257.2 | 102 | 9 |  |
| t-ZR-D5 | [M+H]^+^ | 357.3/225.2 | 116 | 17 |  |
| t-ZR | [M+H]^+^ | 352.2/220.3 | 120 | 9 |  |
| DHZR | [M+H]^+^ | 354,2/222,1 | 124 | 14 |  |
| c-ZR | [M+H]^+^ | 352.2/220.3 | 120 | 9 |  |
| IAAsp | [M+H]^+^ | 291.2/130.1 | 54 | 25 |  |
| IP | [M+H]^+^ | 204.1/148.3 | 90 | 9 | 7.4 |
| IAGlu | [M+H]^+^ | 305.2/130.1 | 58 | 29 |  |
| KR | [M+H]^+^ | 348.2/216.3 | 116 | 9 | 8.5 |
| SA-D4 | [M+H]^+^ | 143.2/125.2 | 80 | 14 |  |
| SA | [M+H]^+^ | 139.2/121.2 | 80 | 14 |  |
| IAA-D5 | [M+H]^+^ | 181.1/135.1 | 38 | 14 |  |
| IAA | [M+H]^+^ | 176.1/130.3 | 51 | 9 |  |
| GA3 | [M-H_2_O+H]^+^ | 329.3/311.3 | 100 | 14 |  |
| GA1-D2 | [M-H_2_O+H]^+^ | 333.3/287.2 | 58 | 9 | 10.3 |
| GA1 | [M-H_2_O+H]^+^ | 331.3/285.3 | 100 | 14 |  |
| ABAGlc | [M-H_2_O+H]^+^ | 409.2/247.1 | 104 | 14 |  |
| GA6 | [M-H_2_O+H]^+^ | 329.3/283.3 | 104 | 14 | 12.4 |
| IPR | [M+H]^+^ | 336.2/204.1 | 124 | 14 |  |
| ABA-D6 | [M-H_2_O+H]^+^ | 253.4/191.3 | 80 | 14 | 16.7 |
| ABA | [M-H_2_O+H]^+^ | 247.4/187.2 | 80 | 14 |  |
| 4Cl-IAA | [M+H]^+^ | 210.4/164 | 80 | 14 |  |
| GA5 | [M-H_2_O+H]^+^ | 285.1/267.1 | 96 | 5 |  |
| JA-D5 | [M+H]^+^ | 216.3/153.2 | 80 | 5 | 18.3 |
| JA | [M+H]^+^ | 211.3/151.2 | 80 | 14 |  |
| 5Cl-IAA | [M+H]^+^ | 210.4/164 | 80 | 14 |  |
| IBA | [M+H]^+^ | 204.1/186.4 | 69 | 9 |  |
| GA7 | [M-H_2_O+H]^+^ | 313.2/223.1 | 104 | 14 | 20.9 |
| GA4-D2 | [M-H_2_O+H]^+^ | 317.3/271.2 | 88 | 9 |  |
| GA4 | [M-H_2_O+H]^+^ | 315.3/269.3 | 100 | 14 |  |
| MeJA | [M+H]^+^ | 225.3/151.2 | 58 | 5 | 21.8 |
| dinorOPDA-D5 | [M+H]^+^ | 270.3/252.2 | 84 | 5 | 23.1 |
| GA9 | [M-H_2_O+H]^+^ | 271.3/225.2 | 136 | 13 |  |
| OPDA | [M+H]^+^ | 293.3/275.2 | 68 | 9 |  |
